# Global Proteome-Wide Analysis of Cysteine S-Nitrosylation in *Toxoplasma gondii*

**DOI:** 10.3390/molecules28217329

**Published:** 2023-10-29

**Authors:** Zexiang Wang, Jia Li, Qianqian Yang, Xiaolin Sun

**Affiliations:** College of Veterinary Medicine, Gansu Agricultural University, Lanzhou 730070, China; lijia@st.gsau.edu.cn (J.L.); sunxl@gsau.edu.cn (X.S.)

**Keywords:** *Toxoplasma gondii*, post-translational modification, cysteine S-nitrosylation, S-nitrosylated proteome

## Abstract

*Toxoplasma gondii* transmits through various routes, rapidly proliferates during acute infection and causes toxoplasmosis, which is an important zoonotic disease in human and veterinary medicine. *T. gondii* can produce nitric oxide and derivatives, and S-nitrosylation contributes to their signaling transduction and post-translation regulation. To date, the S-nitrosylation proteome of *T. gondii* remains mystery. In this study, we reported the first S-nitrosylated proteome of *T. gondii* using mass spectrometry in combination with resin-assisted enrichment. We found that 637 proteins were S-nitrosylated, more than half of which were localized in the nucleus or cytoplasm. Motif analysis identified seven motifs. Of these motifs, five and two contained lysine and isoleucine, respectively. Gene Ontology enrichment revealed that S-nitrosylated proteins were primarily located in the inner membrane of mitochondria and other organelles. These S-nitrosylated proteins participated in diverse biological and metabolic processes, including organic acid binding, carboxylic acid binding ribose and phosphate biosynthesis. *T. gondii* S-nitrosylated proteins significantly contributed to glycolysis/gluconeogenesis and aminoacyl-tRNA biosynthesis. Moreover, 27 ribosomal proteins and 11 microneme proteins were identified as S-nitrosylated proteins, suggesting that proteins in the ribosome and microneme were predominantly S-nitrosylated. Protein–protein interaction analysis identified three subnetworks with high-relevancy ribosome, RNA transport and chaperonin complex components. These results imply that S-nitrosylated proteins of *T. gondii* are associated with protein translation in the ribosome, gene transcription, invasion and proliferation of *T. gondii*. Our research is the first to identify the S-nitrosylated proteomic profile of *T. gondii* and will provide direction to the ongoing investigation of the functions of S-nitrosylated proteins in *T. gondii*.

## 1. Introduction

Toxoplasmosis, caused by parasitic protozoa *Toxoplasma gondii,* is an important zoonotic disease in human and veterinary medicine and has been reported worldwide [1,2]. In some countries, over 60% of the population has either acquired *T. gondii* infection or was seropositive for *T. gondii* [1,3]. The *T. gondii* infection rate is influenced by several factors, such as environmental conditions and dietary habits [1]. People who eat undercooked or raw meat have high infection rates, and cats living in rural districts or stray cats are more susceptible to *T. gondii* infection and excretion oocysts than pet cats [2,3,4]. Sheep and pig meats are at a higher risk of contamination with tissue cysts than cattle meat. Oocysts excreted by cats are unnecessary for its contamination [1,5,6,7]. 

The genus *Toxoplasma* includes only one species, which is grouped into three dominant genotypes: Types I, II and III. The virulent Type I genotype can cause 100% mortality and the median lethal dose (LD50) for Type I is one parasite. Types II and III cause intermediate or no mortality in mice irrespective of the inoculating dose [8,9]. *T. gondii* can infect cells at the sporozoite, bradyzoite and tachyzoite stages [1,10]. Sporozoites are only present in the intestinal cells of definitive hosts, including members of the family Felidae, such as the domestic cat [11,12,13]. *T. gondii* are found in bradyzoite and tachyzoite forms in intermediate hosts who acquire the infection via various routes, such as intake of food and water contaminated with sporulated oocysts, ingestion of raw or underdone meat contaminated with viable tissue cysts, congenital transmission, transplantation of organs containing tachyzoites or cysts and blood transfusion [1,11]. The slowly replicating bradyzoites primarily cause chronic infections, whereas the rapidly replicating tachyzoites cause acute infections [1,14].

Similar to that in other protozoans, *T. gondii* produces nitric oxide (NO) and NO derivatives, indicating the presence of a natural or inducible NO synthase (NOS) in *T. gondii* [15,16]. S-nitrosylation was considered as the extensive mechanism of signaling transduction participated by NO and NO derivatives, in which the S-nitrosylation of cysteine was induced by stable and soluble peroxynitrite (H_2_O_2_ + NO_2_^−^ → ONOO^−^ + H_2_O) resulting from NO and ROS [17,18,19,20,21]. This modification was catalyzed by nitrosylase and denitrosylase to add and remove the nitrosyl group from the substrate, respectively [22,23]. In addition, S-nitrosylation indirectly mediated other modifications, such as disulfide bonds formation and phosphorylation [24,25,26]. Therefore, protein S-nitrosylation not only contributes to cell signaling, but also to protein post-translation regulation [21].

In protozoa, proteome-wide studies on protein S-nitrosylation are limited. Mule et al. used mass spectrometry combined with resin-assisted enrichment and identified and qualified S-nitrosylated proteins from *Trypanosoma cruzi* trypomastigotes and trypomastigotes incubated with the extracellular matrix (ECM). They provided the first S-nitrosylation proteome data in *T. cruzi* and indicated the modulatory function of protein S-nitrosylation on extracellular matrix incubation before *T. cruzi* infection of the mammalian host [21]. The S-nitrosylated proteome of *T. gondii* is still a mystery. In this study, the S-nitrosylation proteome profile of tachyzoites of the *T. gondii* RH strain was identified using mass spectrometry coupled with resin-assisted enrichment, and the function of S-nitrosylated proteins was analyzed using bioinformatics tools. Our results demonstrate the presence of S-nitrosylation in the proteome of *T. gondii* and lay the foundation for studies on the role of S-nitrosylated proteins in *T. gondii*.

## 2. Results

### 2.1. Global Detection of S-Nitrosylated Sites on T. gondii Proteins

**Figure 1:**
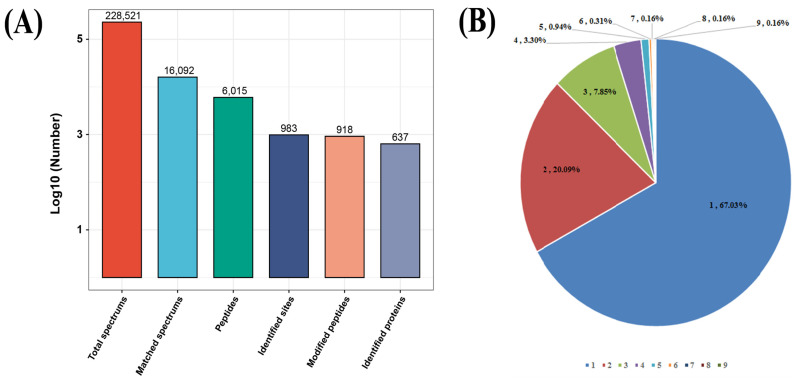
Properties of S-nitrosylated peptides in *Toxoplasma gondii*. (**A**) Number of S-nitrosylated peptides, identified proteins and S-nitrosylated sites. (**B**) Distribution of number of S-nitrosylated sites indicating that the most S-nitrosylated peptides possess a single S-nitrosylated site.

The total protein obtained from *T. gondii* tachyzoites of the RH strain were digested and S-nitrosylated peptides were enriched using the iodoTMT reagent and anti-TMT antibody. The enriched peptides were identified using an easy n-LC 1200 high-performance liquid chromatography (HPLC) system. A total of 918 unique S-nitrosylated peptides, 983 nonredundant S-nitrosylated sites and 637 S-nitrosylated proteins were identified with FDR (false-discovery rate) (Figure 1A, Table S1 in Supplementary Materials). Among these 637 S-nitrosylated proteins, 427 (67.03%) and 128 (~20.09%) were S-nitrosylated at one cysteine residue and two cysteine residues, respectively (Figure 1B). Interestingly, 12.87% (82 out of 637) S-nitrosylated proteins were S-nitrosylated at multiple cysteine residues/sites (more than two S-nitrosylated sites). The prediction of S-nitrosylation sites and S-nitrosylated proteins in *T. gondii* using GPS SNO 1.0 software identified 5587 S-nitrosylated proteins (Table S2). The ratio between predicted vs. identified S-nitrosylated proteins was 8.77. A wide range of S-nitrosylated proteins (450 out of 637) characterized in this study were potential S-nitrosylated proteins as suggested by the GPS SNO 1.0 software, which accounted for 70.64% of total S-nitrosylated proteins characterized in this study.

### 2.2. Motifs of S-Nitrosylated Peptides

**Figure 2:**
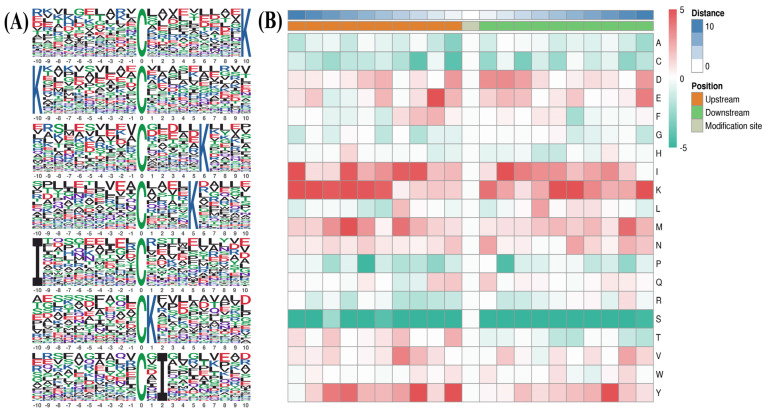
(**A**) Identification of S-nitrosylation-specific motifs using Motif-X. Sequence motif logos denoting identified S-nitrosylated site and composition position-specific amino acids adjacent to S-nitrosylated sites. The frequency of an amino acid emerging in specific positions is represented by the height of its logos. Acidic and basic residues are shown in blue and red, respectively. (**B**) Heat map showing amino acids at positions −10 to +10 from the S-nitrosylated lysine residue. Red and green indicate high and low frequencies, respectively.

Motif-X was used to compare position-specific frequencies among 10 amino acids upstream and downstream of all S-nitrosylated cysteine (Cys) residues. Consensus sequence analysis was conducted across all 637 S-nitrosylated proteins identified. As shown in Figure 2A, we obtained seven putative consensus sequences, including CXXXXXXXXXK, KXXXXXXXXXC, CXXXXXK, CXXXXK, IXXXXXXXXXC, CK and CXI (where C indicates the Cys S-nitrosylated site, X represents any amino acid residue, K indicates lysine acid residue and I represents isoleucine acid residue), which matched 76, 72, 61, 58, 43, 41 and 38 S-nitrosylated protein sites identified in our study (Table 1). In addition, we used MoMo software (Version 5.5.4) and hierarchical cluster analysis to investigate the frequency of neighboring amino acid adjacent to S-nitrosylated Cys residues. The highest frequency of acid residues adjacent to S-nitrosylated Cys residues contained three hydrophilic amino acids (K positions ranging from −10 to −9, −7, +10, and +6 to +5; Y at position ranging from −2 to −1, and +8; E at −2) and two hydrophobic amino acids (I at −10 and +2; M at +7) (Figure 2B).

### 2.3. Functional Annotation of S-Nitrosylated Proteins

**Figure 3:**
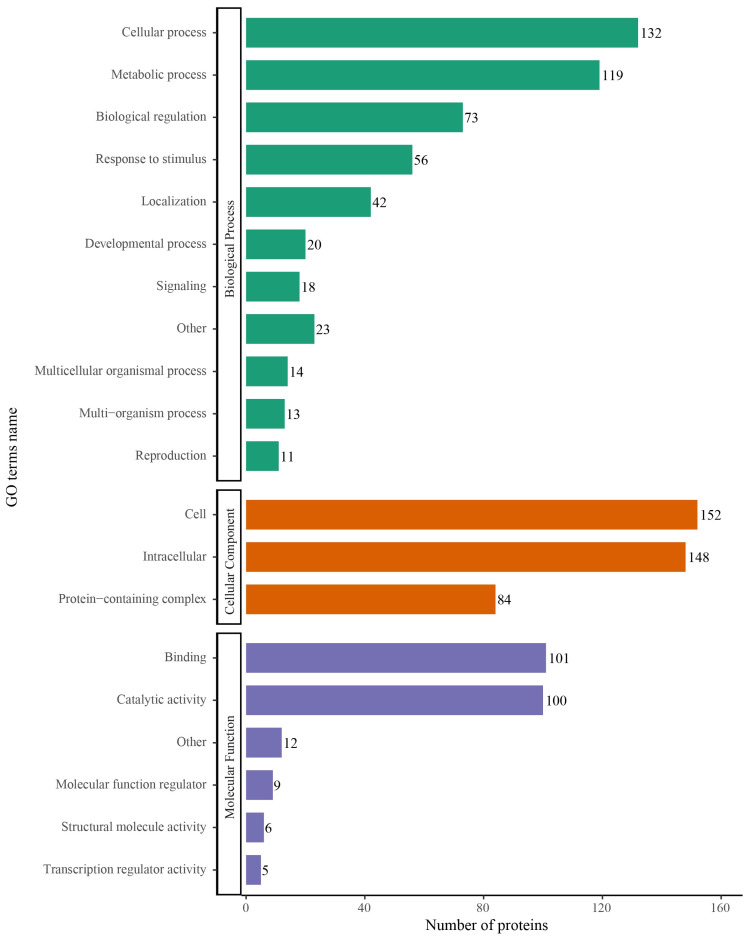
Gene Ontology (GO) classification of S-nitrosylated proteins in *Toxoplasma. gondii*. GO classification of characterized S-nitrosylated proteins based on biological process, cellular component and molecular function.

**Figure 4:**
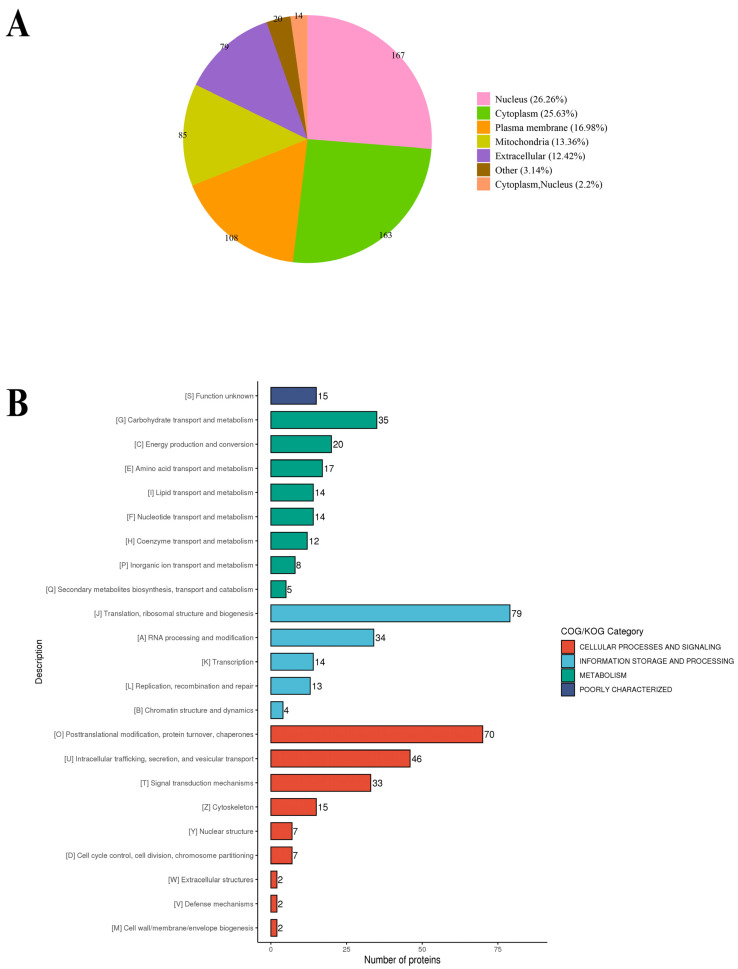
(**A**) Gene Ontology classification of characterized S-nitrosylated proteins based on subcellular localization. (**B**) COG/KOG analysis of S-nitrosylated proteins in *Toxoplasma. gondii*.

To elucidate the primary role of S-nitrosylated proteins in *T. gondii*, Gene Ontology (GO) functional classification was conducted and categorized using enriched GO terms at the second level in the three GO classes: biological process, cellular component and molecular function. In the biological process category, 132, 119 and 73 S-nitrosylated proteins were involved in the cellular process, metabolic process and biological regulation, respectively, accounting for 48.14% of all identified S-nitrosylated proteins in *T. gondii* (Figure 3). In the cellular component category, 152, 148 and 84 S-nitrosylated proteins were involved in the cell, intracellular and protein-containing complex, respectively (Figure 3). In the molecular function category, 101 and 110 S-nitrosylated proteins were associated with binding and catalytic activity, respectively (Figure 3). WoLF PSORT was used to analysis the subcellular localization of the identified S-nitrosylated proteins [27,28]. The S-nitrosylated proteins were mainly present in the nucleus (26.26%), cytoplasm (25.63%), plasma membrane (16.98%), mitochondria (13.36%) and extracellular region (12.42%) (Figure 4A). In COG/KOG analysis, most S-nitrosylated proteins were grouped into translation, ribosomal structure and biogenesis, post-translational modification, protein turnover, chaperones, intracellular trafficking, secretion and vesicular transport, and carbohydrate transport and metabolism (Figure 4B).

### 2.4. Functional Enrichment Analysis of S-Nitrosylated Proteins in T. gondii

**Figure 5:**
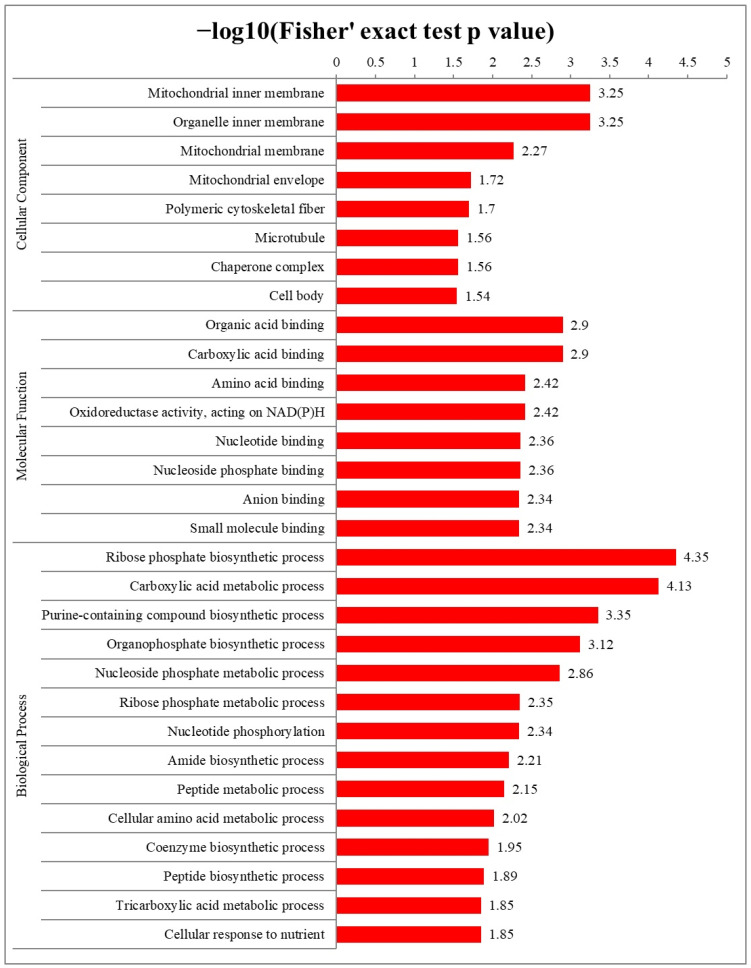
Gene Ontology (GO) enrichment analysis of S-nitrosylated proteins in *Toxoplasma gondii* according to cellular component, molecular function and biological process. The *y*- and *x*-axis denote enriched terms and Rich factor values of GO terms, respectively. Rich factor refers to S-nitrosylated proteins in GO terms divided by total S-nitrosylated proteins. A high degree of enrichment is indicated by high Rich factor values.

**Figure 6:**
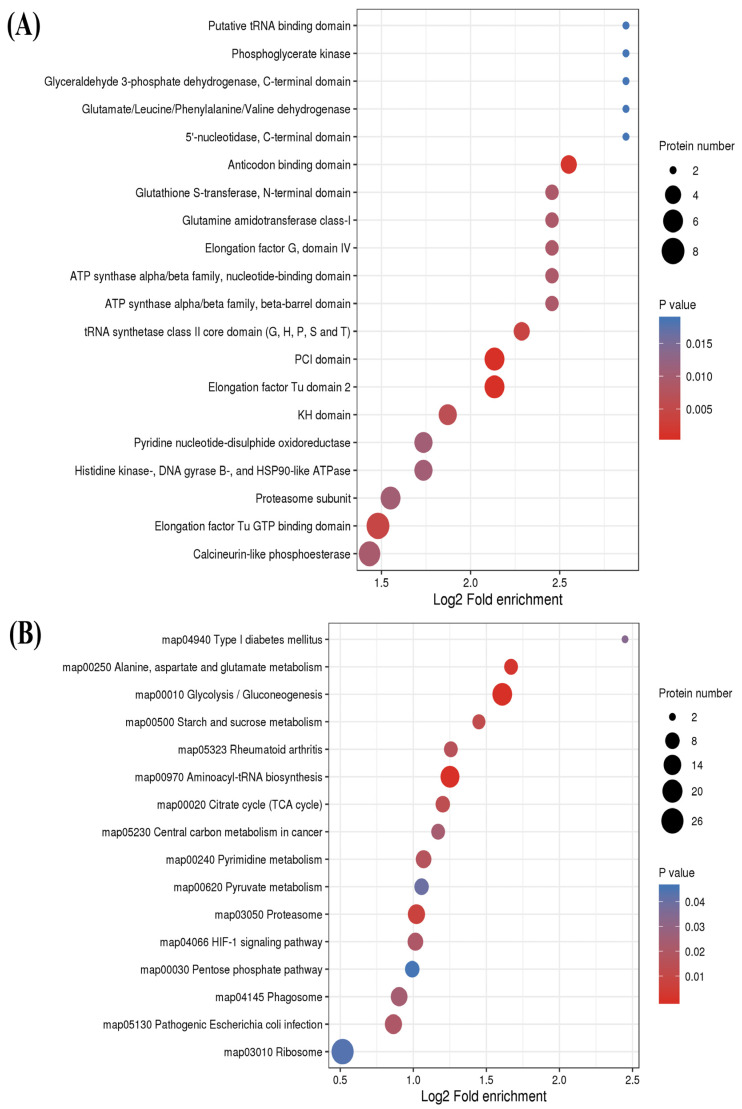
(**A**) Protein domain enrichment analysis of S-nitrosylated proteins in *Toxoplasma gondii*. The *y*- and *x*-axis represent significantly enriched protein domain categories and Rich factor of the protein domain, respectively. Rich factor refers to S-nitrosylated proteins harboring the protein domain divided by total S-nitrosylated proteins. A high degree of enrichment is indicated by high Rich factor values. The color and size of a node corresponding to a protein domain represent the *p* value and quantity of S-nitrosylated proteins in a protein domain. (**B**) Kyoto Encyclopedia of Genes and Genomes (KEGG) pathway analysis of S-nitrosylated proteins in *T. gondii*. The *y*- and *x*-axis represent the KEGG pathways significantly enriched values and Rich factors of the pathways, respectively. Rich factor refers to the ratio of S-nitrosylated proteins in the pathway to the total S-nitrosylated proteins. Greater degrees of enrichment are indicated by higher Rich factor values. The color and size of a node corresponding to a pathway represent the *p* value and quantity of S-nitrosylated proteins in that pathway.

To comprehensively understand the functions of S-nitrosylated proteins, GO enrichment and Kyoto Encyclopedia of Genes and Genomes (KEGG) pathway enrichment analyses were conducted in *T. gondii*. The results of the cellular component category in the GO enrichment analysis revealed that S-nitrosylated proteins were primarily located in the inner membrane of mitochondria and other organelle (Figure 5 and Figure S1). Organic acid binding and carboxylic acid binding were the two most significantly enriched categories under the molecular function. The ribose phosphate biosynthetic process was mostly enriched in biological process terms (Figure 5, Figures S2 and S3). Thereafter, the protein domain database was used to perform enriched protein domain analysis, which revealed that S-nitrosylated proteins were present in 25 domains. As shown in Figure 6A, S-nitrosylated proteins were significantly enriched in the PCI domain and elongation factor Tu domain 2 followed by anticodon binding and tRNA synthetase class II core (G, H, P, S and T) domains. The KEGG pathway analysis indicated that S-nitrosylated proteins mainly participated in glycolysis/gluconeogenesis and aminoacyl-tRNA biosynthesis. It is noteworthy that 19 and 17 S-nitrosylated proteins participated in glycolysis/gluconeogenesis and aminoacyl-tRNA biosynthesis pathways, respectively (Figure 6B).

### 2.5. Cysteine S-Nitrosylation of the Ribosome

Ribosomes are the site of protein synthesis, and ribosomal proteins are involved in protein translation and nuclease, transcription factor and cell signal regulation [29]. Studies have shown that >50% of the proteome of a protozoan comprises ribosomal proteins [29]. The ribosomes of *T. gondii* contain 79 ribosomal proteins [30,31]. Genes encoding ribosomal proteins are randomly located in the *T. gondii* genome, wherein 71 ribosomal proteins, there is a single locus across and eight loci paired at four locations in a head-to-head arrangement [30,31]. Of the 79 ribosomal proteins in *T. gondii*, 27 were *S*-nitrosylated and up to 35% of ribosomal proteins were S-nitrosylated (Table 2).

### 2.6. Cysteine S-Nitrosylation of Microneme Proteins in T. gondii

Micronemes, rhoptries and dense granules are three distinct regulated secretory organelles that which secrete various secretory proteins called micronemal proteins (MICs), rhoptry proteins (ROPs) and dense granule proteins (GRAs). It was suggested that MICs facilitate parasite attachment to host cells, ROPs enhance parasite vacuole formation and GRAs likely promote the replication of intracellular parasites [32]. In *T. gondii*, a number of secretory proteins (MICs, ROPs and GRAs) are considered as virulence-related *p* [30,33,34,35]. In our study, 22 secretory proteins, including 11 MICs, 10 ROPs and 1 GRA, were identified as S-nitrosylated proteins (Table 3). *T. gondii* micronemes contain approximately 20 types of MICs [36,37]. The findings of this study show that >50% of MICs are S-nitrosylated.

### 2.7. Protein–Protein Interaction Networks of S-Nitrosylated Proteins in T. gondii

**Figure 7:**
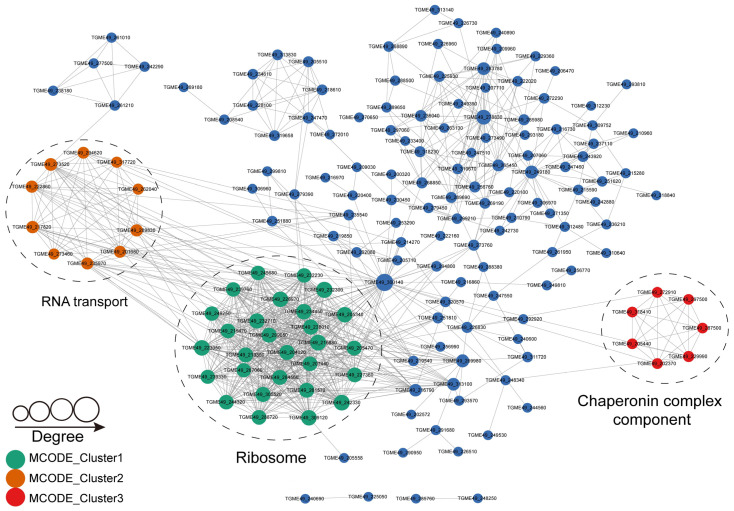
Protein–protein interaction networks of S-nitrosylated proteins in *T. gondii* (PPI, combined score ≥ 0.9). Nodes denote S-nitrosylated proteins and edges represent interactors of S-nitrosylated proteins. The color of an edge refers to the combined score of interactors.

To identify the critical biological processes influenced by S-nitrosylation in *T. gondii*, we conducted protein–protein interaction (PPI) analysis using the STRING database (Search Tool for the Retrieval of Interacting Genes/Protein, ver. 11.0, Wellcome Genome Campus, Hinxton, Cambridgeshire, UK, http://string-db.org/ accessed on 16 October 2023). We identified 204 S-nitrosylated proteins that were mapped to the 1607 protein–protein interactors. The S-nitrosylated PPI networks with high confidence (confidence score > 0.9) were identified by visualizing them using Cytoscape software (ver. 3.2.0, National Institute of General Medical Sciences, National Institutes of Health, Bethesda, MD, USA), which included 204 S-nitrosylated proteins mapped to 818 protein–protein interactors (Figure 7). Subsequently, several highly related subnetworks of S-nitrosylated proteins were identified using MCODE (Minimal Common Oncology Data Elements), including ribosome, RNA transport and chaperonin complex components (Figure 7), which were in agreement with the results obtained in COG/KOG analysis, suggesting that these processes play important roles in shaping the proteomic landscape of S-nitrosylation in *T. gondii*.

## 3. Discussion

NO is a critical and versatile molecule in the immune system that mediates redox regulation in eukaryotic cells when they are infected by unicellular parasites, including *T. gondii* [38]. Among protein PTMs, which regulate various mechanisms involved in cellular survival, growth and the biochemical network, S-nitrosylation is a versatile post-translational modification involved in several processes, such as RNS detoxification and cell cycle regulation [39]. S-nitrosylation affects protein function followed by an alteration in protein conformation, interaction between proteins, activity of enzymes or cellular location [40]. In this study, we identified the S-nitro proteome, a novel type of protein modification, in *T. gondii*.

It was infeasible to directly obtain pure and viable *T. gondii* tachyzoites from mouse peritoneal exudates or cell cultures, so it was crucial that we separated and purified tachyzoites in the process of conducting *T. gondii* research [41,42]. Human foreskin fibroblast (HFF) cells have long be used as the primary cell line to culture *T. gondii* in vitro [43]. It was reported that a confluent monolayer of HFF cells was lysed within 2 and 3 days post infection with ~5 × 10^6^ tachyzoites and that over 90% of tachyzoites were released from HFF cells and were located outside the cell in the infected HFF monolayer [43]. So, extracellular tachyzoites were harvested after release from HFF cells 3~4 days post infection with ~5 × 10^6^ tachyzoites in our study. Among four methods usually used for the purification of *T. gondii* tachyzoites cultured in vivo and in vitro, purification using a 3 μm filter membrane exhibited less contamination with host cells and high yield of tachyzoites [42,44]. This method for the purification of *T. gondii* tachyzoites had been widely used in PTMs proteomic profiling of *T. gondii* including identification of malonylated proteins and crotonylated proteins [27,45]. In the present study, harvested extracellular tachyzoites were also purified using a 3 μm filter membrane to eliminate cell debris. Highly purified tachyzoites without cell debris observed under a microscope were harvested for protein S-nitrosylation profiling.

Because of challenges concerned with the isolation, site-specific identification and quantification of labile and dynamic protein post-translational modifications, our overall understanding of cysteine-based reversible modifications, including S-nitrosylation, remains limited [46]. With regard to the profile of different reversible cysteine modifications together with S-nitrosylation, the sample pre-processing procedure was critical. Guo et al. optimized comprehensive conditions for selective reduction and possible negative or positive controls of specific types of reversible modifications, as well as the blocking of free thiols [46]. A number of previous reports have demonstrated that ascorbate is commonly applied to reduce S-nitrosylation [47,48]. This is consistent with our present study. Su et al. compared the specificity and coverage of enriched cysteine-containing peptides at both protein- and peptide-level enrichment, which showed that peptide-level enrichment has an advantage in the field of resin-binding capacity and is preferred when the amount of starting materials is large or the proteins are difficult to dissolve [47,48]. So, a peptide-level enrichment method was applied by most research studies and plentiful S-nitrosylated sites were identified [21,49,50,51]. In the isolation and purification of biochemical preparations or assays, DTT is widely used and forms a stable six-membered ring with an internal disulfide bond to disrupt S-S bonds formation and prevent protein aggregation [52,53]. In addition, DTT is also a commonly used reagent for total oxidation in the pre-processing procedure of total reversible cysteine modifications, and is able to prevent free thiols from oxidation during the profiling of S-nitrosylation [46,52]. Therefore, DTT is also used in the pretreatment process of the profiling of reversible cysteine modifications, including S-nitrosylation, as well as in our study [46,48].

Notably, *T. gondii* phosphofructokinase PFKII was the most nitrosylated protein (with nine nitrosylated sites), and was dispensable for tachyzoite growth or virulence [54]. PFKII-depleted parasites exhibited slower proliferation than PFKII-expressing parasites, whereas normal levels of ATP, a decrease in the flux of the glycolysis and tricarboxylic acid cycle, pyrophosphate accumulation and a reduction in protein synthesis in PFKII-depleted parasites proved by metabolic analyses suggested that PFKII plays a critical role in the maintenance of pyrophosphate homeostasis in *T. gondii* [54]. The second highly nitrosylated protein was anonymous antigen-1 (TGME49_312630), which was nitrosylated at eight sites. The function of this protein is unknown. Lactate dehydrogenase (LDH)1, harboring seven nitrosylated sites, was the third most nitrosylated protein. LDH1 is one of two LDH isoforms that catalyzes the interconversion of pyruvate and lactate to supply energy during anaerobic growth in *T. gondii* [55,56,57]. The growth rates of LDH1 or LDH2 knockdown parasites in either the tachyzoite or bradyzoite stages were different from those of the parental parasites, and differentiation processes in LDH1 or LDH2 knockdown parasites were downregulated when the parasites were grown under in vitro conditions. This indicates that LDH expression is important for the cell cycle of *T. gondii* [58]. The deletion of LDH1 in *T. gondii* not only displayed a reduction in acute parasite virulence and impairment of bradyzoite differentiation in vitro, but also decreased chronic-stage cyst burdens in vivo [55]. Thus, it can be inferred that *Tg* LDH1 is a critical regulator of virulence, bradyzoite differentiation and chronic infection in *T. gondii* [55].

Of the seven conserved motifs identified across the 637 S-nitrosylated cysteines using Motif-X, five motifs were basic amino acid lysine (K) and two were isoleucine (I). Studies on S-nitrosylation sites in proteins are limited; therefore, the consensus motif is unclear. Isabel Pérez-Mato et al. demonstrated that protein S-nitrosylation is governed by basic and acidic amino acids surrounding target cysteines of S-nitrosylated proteins [57]. Our finding suggests that the basic amino acid surrounding the target cysteines is possibly the major regulator of protein S-nitrosylation in *T. gondii*. The motif CXXXXXK characterized in this study possessed lysine at position +6 from the S-nitrosylated site. This lysine motif was also identified in the S-nitrosylated proteome of HeLa cell lysates and nuclear extracts from rat cortical neurons [59,60]. Theoretically, a linear distance between lysine and cysteine <6 Å intensified the nucleophilicity of the thiol group, making the cysteine more susceptible to nitrosylation, whereas the theoretical linear distance from lysine at position +6 from the S-nitrosylated site to cysteine was as far as ~20 Å [58,59]. It is reasonable to speculate that certain structural conformations of lysine present at +6 close to the target cysteine enhanced the vulnerability of cysteine to nitrosylation [58,59]. It is generally considered that S-nitrosylation is prone to be catalyzed by acidic amino acid and that it occurs in hydrophobic conditions, which stabilize S-nitrosylation through the sequestration of radical species and hydrolysis impedance of the process [53,60,61,62,63]. Combined characteristic acidic amino acid with basic lysine contained among the S-nitrosylation motifs reflected that protein nitrosylation was an indirect acid–base catalysis beyond direct acid–base catalysis [59]. Thus, the protein S-nitrosylation of *T. gondii* involves indirect acid–base catalysis or basic amino acid catalysis by motifs adjacent to the target cysteine.

Glycolysis/gluconeogenesis was the most significant pathway identified in the KEGG pathway enrichment analysis of protein S-nitrosylation in *T. gondii*. The *S*-nitrosylation of proteins involved in glycolysis seems to be a common process among protozoa. In *Plasmodium falciparum*, 11 glycolytic proteins were *S*-nitrosylated, reaching 3.4% (11 out of 319) of the total S-nitrosylated proteins [64]. In *P. falciparum*, the inhibition of S-nitrosylation at target site cysteine 153 of glyceraldehyde-3-phosphate dehydrogenase (GAPDH) remarkedly reduced the activity of PfGAPDH [64]. PfGAPDH may act as a signaling switch in *P. falciparum* for tolerance to nitrosative stress. Furthermore, the glycolysis pathway was indicated to be sensitive to redox modulation by NO [64]. A high-throughput proteomic analysis of S-nitrosylated proteins detected using resin-assisted capture in the gastrointestinal protozoan parasite *Entamoeba histolytica* revealed 14 proteins that participated in energy metabolism. Most of these proteins were S-nitrosylated proteins that were involved in glycolysis [65]. Interestingly, the GAPDH of *Entamoeba histolytica* was also an S-nitrosylated protein, which was similar to that of *P. falciparum* [64,65]. S-nitrosylation-modified proteins are also present in *Trichomonas vaginalis*. Protein nitrosylation was shown to regulate glycolysis in *T. vaginalis* and the number of glycolytic enzymes modified by SNO accounted for 17% of all S-nitrosylated proteins in *T. vaginalis*. The most representative LDH (TvLDH), one of the glycolytic enzymes, was also modified by S-nitrosylation. Adaption to the iron-deficient environment by *T. vaginalis* is dependent on the compensatory energy and nicotinamide adenine dinucleotide recycling provided by the S-nitrosylated TvLDH [65]. Accordingly, it is reasonable to assume that most S-nitrosylated proteins in *T. gondii* are enriched in glycolysis/gluconeogenesis.

PPI analysis performed by Minimal Common Oncology Data Elements identified three subnetworks with high relevancy: ribosome, RNA transport and chaperonin complex components. Studies have demonstrated that ribosomal proteins function as transcription factors, nucleases and regulators of cell signaling and also engage in protein translation [66,67]. Therefore, the percentage of ribosomal proteins in the SNO proteome is high [45,67,68,69]. The proportion of ribosomal proteins modified by SNO among the total number of S-nitrosylated protein was as high as 47% in *T. vaginalis* [65]. Our study characterized 27 *S*-nitrosylated ribosomal proteins, indicating that ribosomal proteins are susceptible to S-nitrosylation (Table 2). S-nitrosylated proteins in *T. cruzi* incubated with or without an extracellular matrix were present in large amounts in ribosomes. Notably, ribosomal proteins were one of the enriched protein complexes, as revealed by PPI network analysis in *T. cruzi* [21]. Consistently, our findings show that S-nitrosylated proteins in *T. gondii* were involved in protein translation in ribosomes. Studies have shown that S-nitrosylated proteins in some protozoa are mainly involved in transcription, such as histones [21,62,65]. It was not expected that RNA transport was the second highest relevant subnetwork in the PPI analysis of the S-nitrosylated proteome in *T. gondii*. Both S-nitrosylation and chaperone regulation majorly affected protein misfolding [63]. S-nitrosylation influenced target proteins involved in some key mechanisms, such as the folding, chaperone-mediated removal and refolding of misfolded proteins to prevent cell damage and accumulation of abnormal proteins [63]. S-nitrosylated chaperones in *T. gondii* formed the third subnetwork, i.e., the chaperonin complex component. The study of the effect of *S*-nitrosylated chaperones in *T. gondii* on its proliferation and virulence can yield interesting results. Among the 22 secretory proteins identified as S-nitrosylated protein in our study, 11 were located in the microneme, accounting for 50% of total MICs reported in the literature [36,37].

MICs play an important role in the initial stages of host cell invasion by *T. gondii* and many types of MICs are effective vaccine candidates against *T. gondii* [70,71]. In addition to nitrosylation, MICs are crotonylated at lysine residues [45]. Ten MICs (MIC1, MIC2, MIC3, MIC4, MIC6, MIC7, MIC8, MIC11, MIC13 and MIC15) are simultaneously crotonylated and nitrosylated. Moreover, several of these MICs are prominent virulent factors, such as MIC1, MIC3, MIC4, MIC6 and MIC8 [33,35]. MIC1, MIC4 and MIC6 assemble into a complex, which is critical for attachment and penetration into host cells during *T. gondii* infection [35]. MIC8 not only functions as an escort of soluble adhesions during invasion, but also participates in the intracellular replication of *T. gondii* [72]. Owing to its ability to generate humoral and cellular immune responses in MIC8-induced mice, it is considered a promising vaccine candidate against acute and chronic toxoplasmosis [45,71,73]. Thus, nitrosylation plays several roles during the invasion of *T. gondii* into the host cell. Further investigation of the function of MIC nitrosylation on the intracellular proliferation and virulence of *T. gondii* will be interesting and insightful.

## 4. Material and Methods

### 4.1. Ethics Approval

All animal experimental procedures were approved by the Animal Administration and Ethics Committee of Gansu Agricultural University (GSAU-Eth-VMC-2023-018), and performed in strict adherence to the Animal Ethics Procedures and Guidelines of the People’s Republic of China. Every effort was made to reduce the suffering of the animals used in the experiments.

### 4.2. Parasite Strains

Tachyzoites of *T. gondii* RH strain used in this study were provided by the State Key Laboratory of Veterinary Etiological Biology, Key Laboratory of Veterinary Parasitology of Gansu Province, Lanzhou Veterinary Research Institute, Chinese Academy of Agricultural Sciences. The RH strain used in our study was owned to type Ⅰ (ToxoDB#10) by applying PCR-restriction fragment length polymorphism (PCR-RFLP) [45,74].

### 4.3. Parasite Collection and Purification

Tachyzoites of *T. gondii* RH strain were maintained by serial passage in human foreskin fibroblast (HFF) monolayers. HFF monolayers were grown in Dulbecco’s modified Eagle’s medium (Gibco, Thermal Scientific, Waltham, MA, USA) containing 10% fetal calf serum (Gibco, Thermal Scientific, Waltham, MA, USA) and 100 U/mL penicillin–streptomycin in a 5% CO_2_ humidified incubator at 37 °C, as described [27,53]. A flask containing the HFF monolayer was infected with ~5 × 10^6^ tachyzoites. The uninfected control HFF monolayer was also set up. A host–parasite ratio of 3:1 was used because it has been shown to yield a large number of parasites [75,76]. After infection was complete, HFF monolayer lysis and tachyzoites growth were examined every day. The infected HFF monolayer group contained free crescent-shaped tachyzoites in the cell culture medium, whereas no tachyzoites was observed in the uninfected HFF monolayer group. *T. gondii* infection of HFF monolayer was also characterized by subjecting the samples collected from the two groups to B1 gene amplification and PCR-RFLP. We found that samples from the uninfected HFF monolayer group did not contain tachyzoites, unlike the infected HFF monolayer group that contained tachyzoites of type I RH strain. In the infected HFF monolayer group, ~90% of tachyzoites were released from HFF cells and were located outside the cell 3~4 d after infection. Then, extracellular *T. gondii* tachyzoites and cell debris were harvested and washed several times with phosphate buffered saline [27,53] and passed through a 25-gauge needle. The parasites were filtered through a 3 μm filter membrane (Millipore) to remove HFF cell debris. This step was repeated three times until all cell debris was removed. The obtained sample was observed under a microscope to ensure that samples contained tachyzoites without cell debris. Purified parasites were centrifuged at 3000 rpm for 15 min and stored at −80 °C until use.

### 4.4. Protein Extraction

Tachyzoite pellets were sonicated for 3 min on ice using a high-intensity ultrasonic processor (Scientz) in lysis buffer (1%SDS, 1% protease inhibitor cocktail, 50 mM IAM, [pH 8.0]) and centrifuged at 12,000× *g* and 4 °C for 15 min in order to remove the remaining debris. Finally, protein concentration in the supernatant was measured using the Bradford assay.

### 4.5. Iodo-TMT Labeling

Briefly, 1 mg of each sample (2 μg/μL) was reduced with 10 mM arsenite at 37 °C for 1 h under dark conditions. After specific reduction, proteins were labeled with iodoTMT reagent 130 (ThermoFisher Scientific, Waltham, MA, USA), according to the manufacturer’s protocol, and incubated for 1 h at room temperature in dark.

### 4.6. Trypsin Digestion

Protein samples were mixed with one volume of precooled acetone vortexed and mixed with four volumes of prechilled acetone for precipitation at −20 °C for 2 h. The protein sample was re-dissolved in 200 mM tetraethyl ammonium bromide (TEAB) and ultrasonically dispersed. Protein samples were digested using trypsin at trypsin-to-protein mass ratio of 1:50 at 37 °C overnight. Then, the protein samples were reduced with dithiothreitol final concentration of 5 mM for 60 min at 37 °C and alkylated with iodoacetamide final concentration of 11 mM for 45 min at room temperature under darkness conditions. The samples were subjected to a second digestion using 1:100 trypsin-to-protein mass ratio for 4 h at 37 °C, desalted on a Strata X SPE column and vacuum-dried.

### 4.7. HPLC Fractionation

The sample was fractionated via high-pH reverse-phase HPLC performed using Agilent 300 Extend C18 column (5 μm particles, 4.6 mm ID and 250 mm length). Briefly, peptides were separated with a gradient of 2–60% acetonitrile (ACN) in 10 mM ammonium bicarbonate pH 10 over 80 min into 80 fractions. Then, the peptides were combined into 6 fractions and dried by vacuum centrifuging.

### 4.8. Resin-Assisted Enrichment of S-Nitrosylated Peptides

Enrichment of S-nitrosylated peptides was conducted as described [21,77] using Anti-TMT Resin (Thermo Scientific, Waltham, MA, USA). The lyophilized peptides were resuspended in 1x TBS, transferred to 50 μL of prewashed Anti-TMT Resin and incubated overnight in the dark at 4 °C with rotation. After overnight incubation, the supernatant was removed and the resin was washed eight times (5 min/wash) with 1 column volume of TBS, and three times with 1 column volume of sterile H_2_O. Finally, the sample was eluted with 4 column volumes of TMT Elution Buffer (50% ACN, 0.4% TFA). The eluted fractions were combined and vacuum-dried. For LC-MS/MS analysis, the resulting peptides were desalted with C18 ZipTips (Millipore, Billerica, MA, USA) according to the manufacturer’s instructions.

### 4.9. LC-MS/MS Analysis of Enriched S-Nitrosylated Peptides

Enriched S-nitrosylated peptides dissolved with buffer A (2% ACN, 0.1% formic acid, FA) were separated using an EASY-nLC 1200 HPLC system and EASY-Spray source (Thermo Scientific, Waltham, MA, USA) with EASY-Spray PepMap RSLC C18 25 cm × 75 μm ID column (Thermo Scientific, Waltham, MA, USA). A linear gradient of buffer B (90% ACN, 0.1% FA) running at a flow rate of 500 nL/min was applied for sample elution. The elution started from 9% buffer B and increased to 23% buffer B. Then, the concentration of buffer B was gradually increased to 35% within 5 min followed by a linear increase in its concentration to 80% for 5 min. Buffer B concentration was maintained at 80% for 2 min and then decreased to 5% within 1 min, followed by maintenance at 5% for 10 min. Finally, the spectra of first-grade MS were acquired during a scan scope of 350 to 1600 m/z in the resolution of 1.2 × 10^6^. The spectra of second-grade MS were obtained with the parameter set up as follows: resolution = 1.5 × 10^5^; maximum injection time = 100 ms; AGC target = 1 × 10^5^. The mode of second-grade MS spectra was high-energy collisional dissociation (HCD) and its normalized collision energy was set to 30 eV. Quality control sample was also included and was analyzed in the same way during the LC-MS/MS analysis of enriched S-nitrosylated peptides.

### 4.10. Data Processing and Peptide Identification

Peptides and proteins were identified using the Proteome Discoverer software version 1.4 (Thermo Scientific, Waltham, MA, USA) combined with Mascot 2.3 search engines (Matrix Science, LDN, UK). The MS/MS spectra were queried against the *Toxoplasma gondii* ME49 strain database (http://www.toxodb.org/common/downloads/release-10.0/TgondiiME49/fasta/data/, accessed on 31 January 2014). Parameters of the search included: peptide mass tolerance = 20 ppm; enzyme = trypsin; fragment mass tolerance = 0.05 Da. Dynamic modifications harbored carbamidomethylation (C), iodoTMTsixplex (C) and methionine oxidation. The criteria applied to filter resulting peptides were false discovery rate of less than 0.01 and a confidence level of 95% were maximum 5%.

### 4.11. Bioinformatics Analysis and Results Presentation

Group-based Prediction System for SNO 1.0 (GPS SNO 1.0, ver. 1.0, available at http://sno.biocuckoo.org/ accessed on 16 October 2023) was used to predict plausible S-nitrosylated proteins harboring S-nitrosylation sites with default high threshold among the proteome data of *T. gondii* [78,79]. To obtain the probable S-nitrosylation motifs of S-nitrosylated peptides characterized in our research, the Motif-X algorithm [80] was utilized to analyze the bias of amino acid residues approximating S-nitrosylation sites and investigate motif consensus sequences for protein S-nitrosylation. The logo-like representation was used to graphically display motifs created in the alignment. Subcellular localization of nitrosylated proteins was predicted by using the WoLF PSORT (ver. 0.2, https://wolfpsort.hgc.jp accessed on 16 October 2023). Subsequently, GO annotation was conducted by applying the UniProt-GOA (http://www.ebi.ac.uk/goa/, accessed on 3 May 2022) database. The InterProScan software (ver. 5.0) was used to annotate protein’s GO function stemming from protein sequence alignment method in case of absence of annotation by UniProt-GOA database. Using GO (ver. 4.0, Open Biological and Biomedical Ontology Foundry, San Francisco, CA, USA, http://www.geneontology.org accessed on 16 October 2023) analysis, S-nitrosylated proteins were categorized into three classes, including molecular function, biological process and cell component. The KEGG (ver. 89.1, Institute for Chemical Research, Kyoto University, Kyoto, Japan, http://www.kegg.jp/kegg/ accessed on 16 October 2023) analysis was performed to annotate S-nitrosylated proteins to KEGG pathways. Meanwhile, InterProScan software (ver.5.0) and InterPro domain database were applied to annotate the protein domains based on protein sequence alignment. The GO/KEGG/domain enrichment analysis of all identified proteins was performed using a two-tailed Fisher’s exact test. The interaction network S-nitrosylated proteins involved were predicted using the STRING database and the PPI network was represented by Cytoscape software. The PPIs whose combined score was >0.9 were subjected to further interaction network analysis.

## 5. Conclusions

In this study, we reported the first S-nitrosylated proteomic profile of *T. gondii* using MS in combination with resin-assisted enrichment. We found 637 S-nitrosylated proteins, more than half of which were localized in the nucleus or cytoplasm. Of the seven identified motifs, five and two contained lysine and isoleucine, respectively. GO enrichment revealed that S-nitrosylated proteins were primarily located in the inner membrane of mitochondria and other organelles. These S-nitrosylated proteins participated in diverse biological and metabolic processes, including organic acid binding, carboxylic acid binding ribose and the phosphate biosynthetic process, indicating that S-nitrosylation contributes to the regulation of the *T. gondii* function. *S*-nitrosylated proteins of *T. gondii* were mainly involved in glycolysis/gluconeogenesis and aminoacyl-tRNA biosynthesis, suggesting that S-nitrosylation plays a crucial role in glycolysis in *T. gondii*. We found that 27 ribosomal proteins and 11 microneme proteins out of 22 secretory proteins were S-nitrosylated proteins, indicating that proteins in the ribosome and microneme were predominantly S-nitrosylated. PPI analysis identified three subnetworks: high-relevancy ribosome, RNA transport and chaperonin complex components. These networks demonstrated that S-nitrosylated proteins in *T. gondii* were associated with protein translation in the ribosome, gene transcription, invasion and proliferation of *T. gondii*. Our results will be a valuable resource for the ongoing investigation of the functions of S-nitrosylation. Comprehensive studies performed to unravel the functions of some prominent S-nitrosylated proteins in *T. gondii* are warranted. A comparative analysis of S-nitrosylated proteomic profiles among bradyzoites, tachyzoites and oocysts in *T. gondii* and between divergent genotypes of *T. gondii* should be performed, which in turn will promote the elucidation of the development and virulence of *T. gondii*.

## Figures and Tables

**Table 1:** Description of S-nitrosylated motifs identified using Motif-X.

No.*	Motif	Motif Score	Foreground		Background		Fold Increase
			Matches	Size	Matches	Size	
1.	xxxxxxxxxxCxxxxxxxxxK	8.72	76	983	4810	131,270	2.1
2.	KxxxxxxxxxCxxxxxxxxxx	8.42	72	907	4731	126,460	2.1
3.	xxxxxxxxxxCxxxxxKxxxx	7.02	61	835	4251	121,729	2.1
4.	xxxxxxxxxxCxxxxKxxxxx	7.20	58	774	4070	117,478	2.2
5.	IxxxxxxxxxCxxxxxxxxxx	6.41	43	716	2897	113,408	2.4
6.	xxxxxxxxxxCKxxxxxxxxx	6.15	41	673	2864	110,511	2.4
7.	xxxxxxxxxxCxIxxxxxxxx	6.86	38	632	2475	107,647	2.6

* Numbers 1–7 represent S-nitrosylated cysteine motifs.

**Table 2:** Ribosomal proteins in proteomics identification of cysteine S-nitrosylated proteins in *Toxoplasma. gondii*.

Gene Product	Gene ID	Number of S-Nitrosylated Site
ribosomal protein RPL3	TGME49_227360	2
ribosomal protein RPL4	TGME49_309120	2
ribosomal protein RPL7A	TGME49_261570	2
ribosomal protein RPL8	TGME49_204020	1
ribosomal protein RPL9	TGME49_284560	1
ribosomal protein RPL10	TGME49_288720	1
ribosomal protein RPL10A	TGME49_215470	3
ribosomal protein RPL14	TGME49_267060	1
ribosomal protein RPL17	TGME49_299050	1
ribosomal protein RPL21	TGME49_245680	2
ribosomal protein RPL22	TGME49_239760	1
ribosomal protein RPL23A	TGME49_238010	1
ribosomal protein RPL24	TGME49_244320	1
ribosomal protein RPL28	TGME49_229250	1
ribosomal protein RPL30	TGME49_232230	1
ribosomal protein RPL35A	TGME49_249250	1
ribosomal protein RPL37	TGME49_239330	1
ribosomal protein RPS2	TGME49_305520	2
ribosomal protein RPS3	TGME49_232300	2
ribosomal protein RPS3A	TGME49_232710	2
ribosomal protein RPS4	TGME49_207440	1
ribosomal protein RPS5	TGME49_242330	2
ribosomal protein RPL10	TGME49_288720	1
ribosomal protein RPS11	TGME49_226970	2
ribosomal protein RPS12	TGME49_205340	4
ribosomal protein RPS15	TGME49_213350	1
ribosomal protein RPS15A	TGME49_234450	1
ribosomal protein RPS20	TGME49_223050	1

**Table 3:** Secretory proteins (MICs, ROPs and GRAs) identified in the cysteine *S*-nitrosylated proteome in *Toxoplasma. gondii*.

Gene Product	Gene ID	Number of S-Nitrosylated Site
MIC1	TGME49_291890	2
MIC2	TGME49_201780	4
MIC3	TGME49_319560	5
MIC4	TGME49_208030	3
MIC6	TGME49_218520	1
MIC7	TGME49_261780	2
MIC8	TGME49_245490	3
MIC11	TGME49_204530	1
MIC13	TGME49_260190	2
MIC15	TGME49_247195	2
MIC17A	TGME49_200250	1
ROP5	TGME49_308090	1
ROP9	TGME49_243730	1
ROP14	TGME49_315220	1
ROP15	TGME49_211290	1
ROP18	TGME49_205250	1
ROP33	TGME49_201130	1
RON2	TGME49_300100	2
RON3	TGME49_223920	4
RON4	TGME49_229010	1
RON5	TGME49_311470	3
GRA12	TGME49_288650	1

## Data Availability

The identifier of ProteomeXchange Consortium depositing mass spectrometry data is PXD046083.

## Supplementary Materials

The following supporting information can be downloaded at: https://www.mdpi.com/article/10.3390/molecules28217329/s1, Figure S1: Bubble chart of GO enrichment analysis of the S-nitrosylated protein according to cellular component in *Toxoplasma gondii.* The *y*-axis denotes the terms of GO enrichment and the *x*-axis represents the Rich factors of the GO terms. Rich factor refers to the ratio of the quantity of S-nitrosylated proteins in the GO terms to the quantity of total S-nitrosylated proteins. Greater degrees of enrichment are indicated by higher Rich factors. Color and size of the node corresponding to GO terms refer to the *p* value and quantity of S-nitrosylated proteins of GO terms; Figure S2: Bubble chart of GO enrichment analysis of the S-nitrosylated protein according to molecular function in *Toxoplasma gondii.* The *y*-axis denotes the terms of GO enrichment and the *x*-axis represents the Rich factors of the GO terms. Rich factor refers to the ratio of the quantity of S-nitrosylated proteins in the GO terms to the quantity of total S-nitrosylated proteins. Greater degrees of enrichment are indicated by higher Rich factors. Color and size of the node corresponding to GO terms refer to the *p* value and quantity of S-nitrosylated proteins of GO terms; Figure S3: Bubble chart of GO enrichment analysis of the S-nitrosylated protein according to biological process in *Toxoplasma gondii.* The *y*-axis denotes the terms of GO enrichment and the *x*-axis represents the Rich factors of the GO terms. Rich factor refers to the ratio of the quantity of S-nitrosylated proteins in the GO terms to the quantity of total S-nitrosylated proteins. Greater degrees of enrichment are indicated by higher Rich factors. Color and size of the node corresponding to GO terms refer to the *p* value and quantity of S-nitrosylated proteins of GO terms; Table S1: List of S-nitrosylated peptides and proteins identified in *Toxoplasma gondii*; Table S2: List of S-nitrosylated sites and proteins predicted using GPS SNO 1.0.
