# Supplementary material for: Global Proteome-Wide Analysis of Cysteine S-Nitrosylation in Toxoplasma gondii
Source: Molecules. 2023 Oct 29;28(21):7329. doi: 10.3390/molecules28217329 (PMC10649196; doi:10.3390/molecules28217329)
Supplement: Supplementary file 1 [file molecules-28-07329-s001.zip › molecules-2626885-Supplementary Figures.pdf]

Supplementary Materials

Global Proteome-wide Analysis of Cysteine S-Nitrosylation in *Toxoplasma gondii*

Zexiang Wang *, Jia Li, Qianqian Yang and Xiaolin Sun

College of Veterinary Medicine, Gansu Agricultural University, Lanzhou 730070, China; lijia@st.gsau.edu.cn (J.L.); yangqq@st.gsau.edu.cn (Q.Y.); sunxl@gsau.edu.cn (X.S.)

* Correspondence: wangzx@gsau.edu.cn

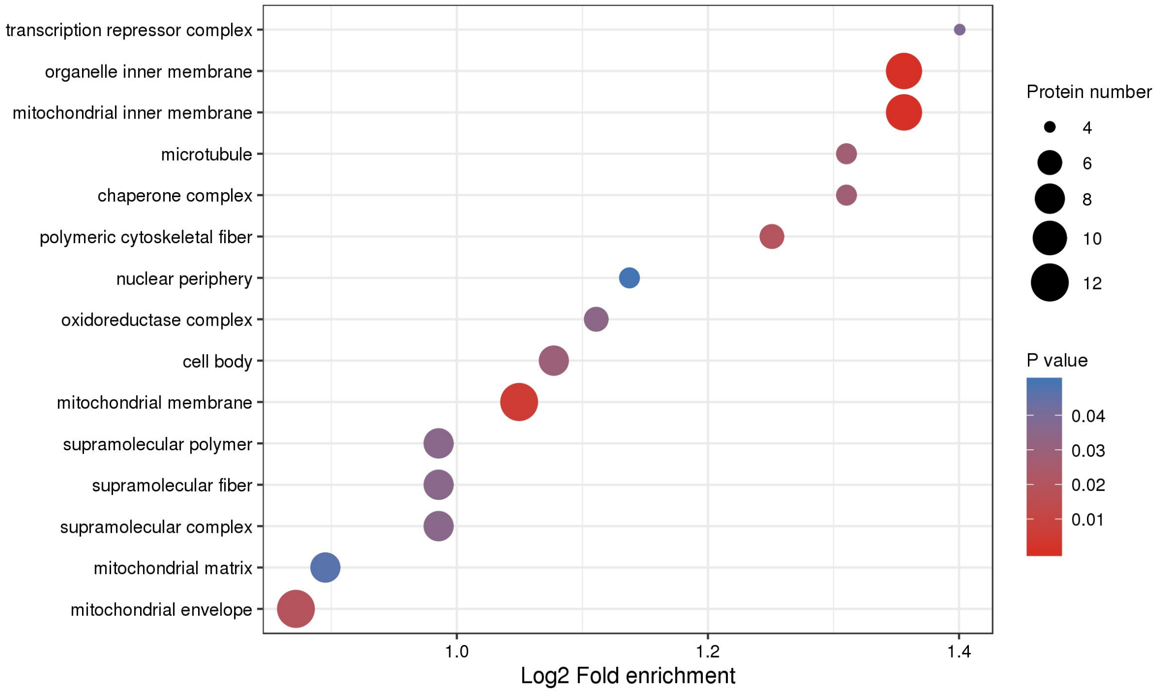

FIGURE S1 | Bubble chart of GO enrichment analysis of the S-nitrosylated protein according to Cellular Component in *Toxoplasma gondii.* The y-axis denotes the terms of GO enrichment and the x-axis represents the rich factors of the GO terms. Rich factor refers to the ratio of the quantity of S-nitrosylated proteins in the GO terms to the quantity of total S-nitrosylated proteins. Greater degrees of enrichment were indicated by higher rich factors. Color and size of the node corresponding to GO terms refers to the *p* value and quantity of S-nitrosylated proteins of GO terms.

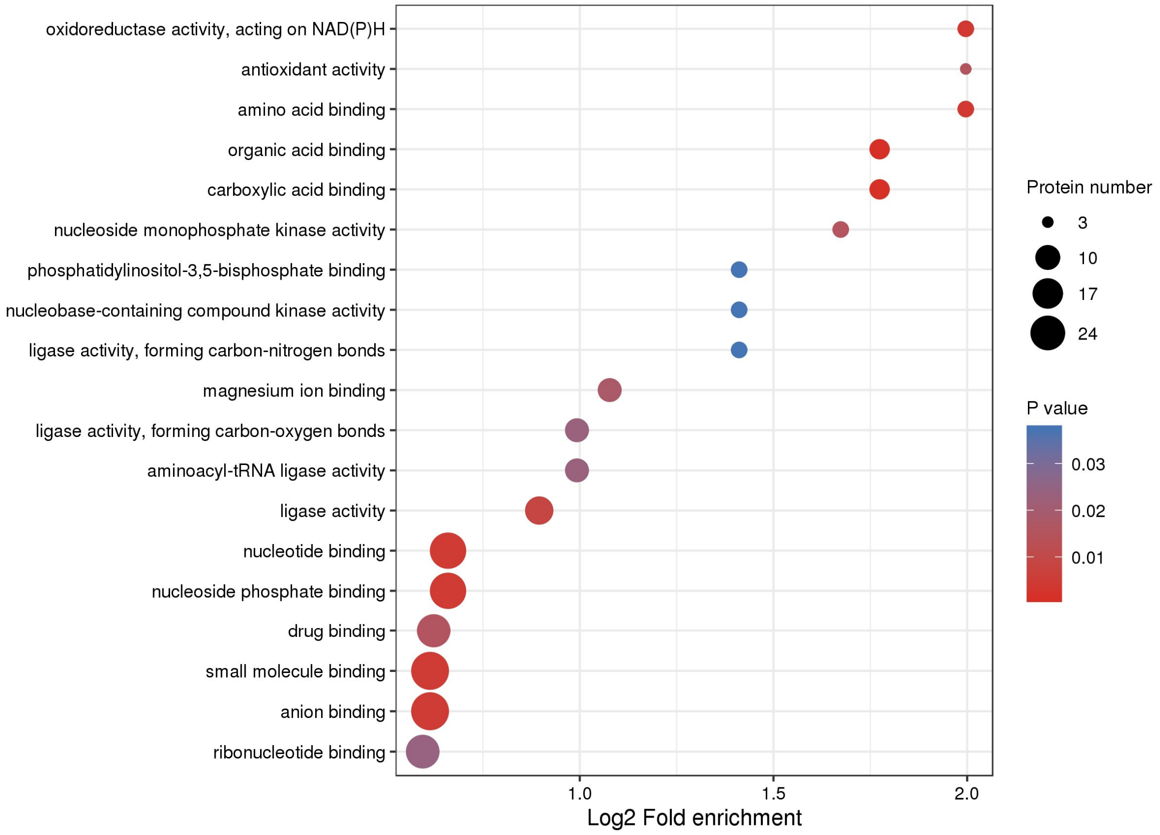

FIGURE S2 | Bubble chart of GO enrichment analysis of the S-nitrosylated protein according to Molecular Function in *Toxoplasma gondii.* The y-axis denotes the terms of GO enrichment and the x-axis represents the rich factors of the GO terms. Rich factor refers to the ratio of the quantity of S-nitrosylated proteins in the GO terms to the quantity of total S-nitrosylated proteins. Greater degrees of enrichment were indicated by higher rich factors. Color and size of the node corresponding to GO terms refers to the *p* value and quantity of S-nitrosylated proteins of GO terms

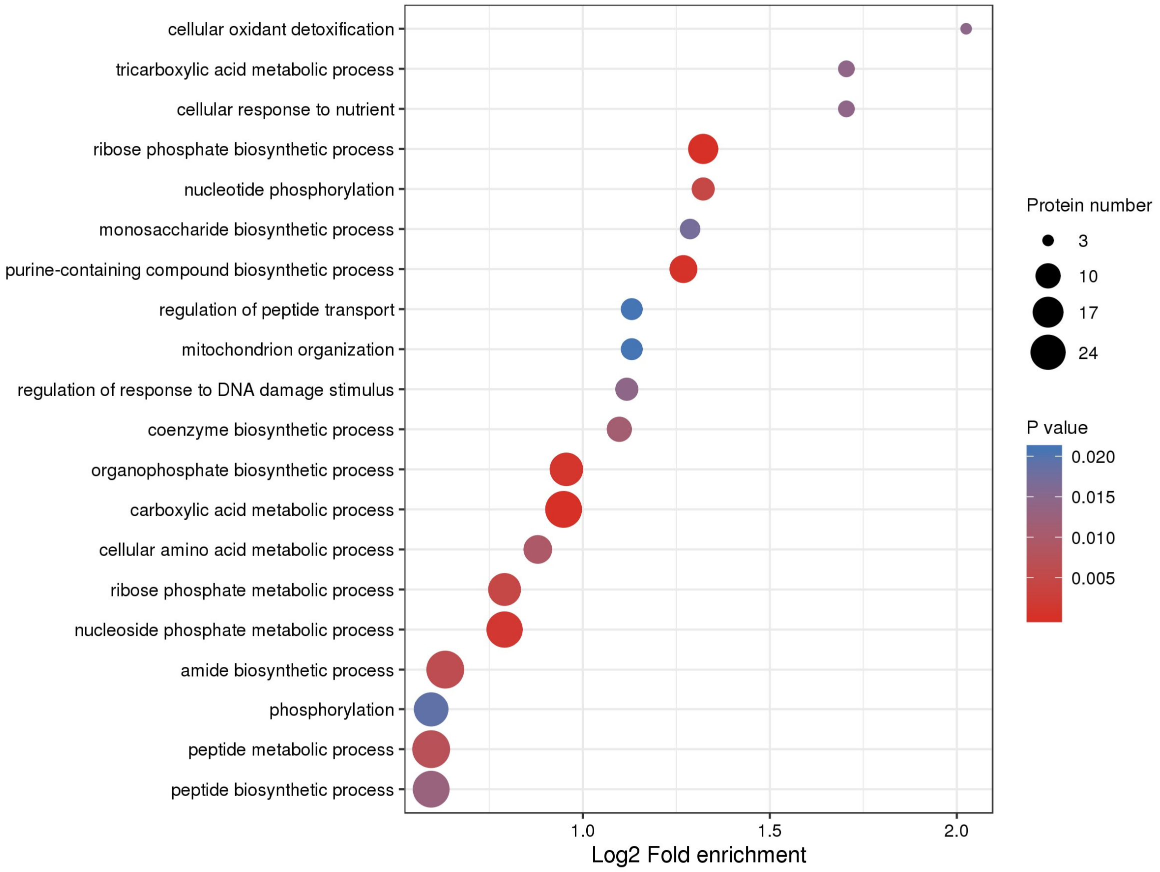

FIGURE S3 | Bubble chart of GO enrichment analysis of the S-nitrosylated protein according to Biological Process in *Toxoplasma gondii.* The y-axis denotes the terms of GO enrichment and the x-axis represents the rich factors of the GO terms. Rich factor refers to the ratio of the quantity of S-nitrosylated proteins in the GO terms to the quantity of total S-nitrosylated proteins. Greater degrees of enrichment were indicated by higher rich factors. Color and size of the node corresponding to GO terms refers to the *p* value and quantity of S-nitrosylated proteins of GO terms.
